# Postnatal LPS Challenge Impacts Escape Learning and Expression of Plasticity Factors *Mmp9* and *Timp1* in Rats: Effects of Repeated Training

**DOI:** 10.1007/s12640-017-9720-2

**Published:** 2017-04-18

**Authors:** Alexander Trofimov, Tatyana Strekalova, Niall Mortimer, Olga Zubareva, Alexander Schwarz, Evgeniy Svirin, Aleksei Umriukhin, Andrei Svistunov, Klaus-Peter Lesch, Victor Klimenko

**Affiliations:** 1 Section of Molecular Psychiatry, Clinical Research Unit of Disorders of Neurodevelopment and Cognition, Centre of Mental Health, University Hospital of Würzburg, University of Würzburg, Margarete-Höppel-Platz 1, 97080 Würzburg, Germany; 2 Laboratory of Neurobiology of the Brain Integrative Functions, I.P. Pavlov Department of Physiology, Institute of Experimental Medicine, Akademika Pavlova 12, 197376 St. Petersburg, Russia; 3 Laboratory of Biomolecular Screening, Institute of Physiologically Active Compounds, Russian Academy of Sciences, Severnii proezd 1, 142432 Chernogolovka, Moscow Region Russia; 4 Department of Neuroscience, Maastricht University, Universiteitssingel 40, NL, 6229 ER Maastricht, Netherlands; 5 Laboratory of Molecular Mechanisms of Neuronal Interactions, I.M. Sechenov Institute of Evolutionary Physiology and Biochemistry, Russian Academy of Sciences, Thorez Avenue 44, 199223 St. Petersburg, Russia; 6 Institute of Molecular Medicine, Laboratory of Psychiatric Neurobiology, I.M. Sechenov First Moscow State Medical University, Trubetskaya 8-2, 119991 Moscow, Russia; 7 Department of Normal Physiology, I.M. Sechenov First Moscow State Medical University, Mokhovaya 11-4, 125009 Moscow, Russia

**Keywords:** Lipopolysaccharide (LPS), MMP-9, TIMP-1, Escape learning, Corticosterone, Rat

## Abstract

Bacterial intoxication associated with inflammatory conditions during development can impair brain functions, in particular evolutionarily novel forms of memory, such as explicit learning. Little is known about the dangers of early-life inflammation on more basic forms of learning, for example, the acquisition of motor escape abilities, which are generally better preserved under pathological conditions. To address this limitation in knowledge, an inflammatory response was elicited in Wistar pups by lipopolysaccharide (LPS) injections (25 μg/kg) on postnatal days P15, P18 and P21. The acquisition of escape behaviour was tested from P77 by active avoidance footshock model and water maze. Open-field behaviour and blood corticosterone levels were also measured. Rat brain tissue was collected from pups 2 h post-injection and from adult rats which either underwent escape training on P77–P81 or remained untrained. mRNA levels of developmental brain plasticity factors MMP-9 and TIMP-1 were investigated in the medial prefrontal cortex and ventral/dorsal hippocampus. LPS-challenged rats displayed moderately deficient escape responses in both memory tests, increased freezing behaviour and, surprisingly, reduced blood cortisol levels. *Mmp9* and *Timp1*, and their ratio to one another, were differentially altered in pups versus adult untrained rats but remained unchanged overall in rats trained in either learning task. Together, our data indicate that systemic pro-inflammatory response during early postnatal development has long-lasting effects, including on the acquisition of motor escape abilities and plasticity factor expression, into adulthood. Our data suggest that altered stress response could possibly mediate these deviations and repeated training might generate positive effects on plasticity under the employed conditions.

**Electronic supplementary material:**

The online version of this article (doi:10.1007/s12640-017-9720-2) contains supplementary material, which is available to authorized users.

## Introduction

Inflammation during the early postnatal period has been implicated in the aetiology of numerous neuropsychiatric conditions including Alzheimer’s disease, schizophrenia, attention-deficit/hyperactivity disorder and autism (Rantakallio et al. 1997; Hornig et al. 1999; Shi et al. 2003; Bilbo and Schwarz 2012). Clinical and pre-clinical studies suggest that compromised brain neural plasticity is a pivotal pathophysiological link to these disorders. Early-life infection can increase levels of cytokines, such as interleukin (IL)-1β, IL-6 and tumour necrosis factor (TNF), and lead to impairments in attention and memory during adolescence and adulthood (de Bont et al. 1993; Aly et al. 2009; Yirmiya and Goshen 2011; Tishkina et al. 2016).

Postnatal administration of lipopolysaccharide (LPS) is a well-established model of the cognitive and behavioural effects of early-life systemic inflammation. Low-dose postnatal LPS administration increases cytokine production in a dose-dependent manner and impairs memory in adulthood (Leonard 2001; Goshen et al. 2007; Donzis and Tronson 2014; Tishkina et al. 2016). The vast majority of studies have focused on evolutionarily late forms of memory, such as explicit learning, a hippocampus-dependent form of memory, which is known to be particularly vulnerable under various pathological conditions. Systemic LPS injection inhibits hippocampal long-term potentiation (LTP) (Vereker et al. 2000) and selectively impairs hippocampus-dependent spatial navigation in the Morris water maze and contextual fear conditioning, whereas cortex-independent auditory-cue fear conditioning remains unaffected (Rachal Pugh et al. 2001; Shaw et al. 2001). Little is known about the effects of early-life inflammation on the development of motor escape abilities, which are generally more preserved under pathological conditions.

Postnatal LPS administration attenuates plasticity-associated factors in the hippocampus and cortex including brain-derived neurotrophic factor (BDNF), nerve growth factor (NGF), neurotrophin-3 (NT-3) and Ca^2+^/calmodulin-dependent protein kinase II (CaMKII), as well as altering TrkA, extracellular signal-regulated kinases and the expression of NMDA receptor subunit NR1 (Lapchak et al. 1993; Raetz and Whitfield 2002; Guan and Fang 2006; Schnydrig et al. 2007; Hennigan et al. 2007; Harré et al. 2008, Calabrese et al. 2014; Dehkordi et al. 2015). Meanwhile, striking differences between early stage and adult molecular and functional organization of the hippocampus question the relevance of these molecular mechanisms, as these adult plasticity markers are poorly expressed during early-life (Travaglia et al. 2016). These concerns are supported by numerous in vitro findings showing opposing, stimulatory effects of pro-inflammatory cytokines on plasticity molecules, such as CaMKII, tyrosine kinases, mitogen-activated protein kinases (MAPKs), protein kinase C (PKC), phosphoinositide-3 kinase (PI3K) and transcription factors such as nuclear factor kappa B (NF-κB) and activator protein 1 (AP-1) (Rosenberg 2002; Wu et al. 2004, 2009).

In the present work, we investigated messenger RNA (mRNA) levels of two functionally related developmental plasticity factors, tissue inhibitor of metalloproteinase 1 (*TIMP-1*) and matrix metalloproteinase 9 (*MMP-9*), after postnatal immune challenge with LPS. These factors help regulate neuronal remodelling and cell-to-cell interactions and are abundantly expressed in the prefrontal cortex and hippocampus (Ethell and Ethell 2007; Janusz et al. 2013; Aujla and Huntley 2014). MMP-9 is expressed in many cell types, including neurons and glia (Reinhard et al. 2015), and multiple brain regions including the prefrontal cortex and hippocampus (Bednarek et al. 2009; Aujla and Huntley 2014). MMP-9 is highly expressed during early brain development and decreases in adulthood (Aujla and Huntley 2014). A major function of MMP-9 is the regulation of cell-to-cell interactions by modifying the extracellular matrix (ECM), cell adhesion molecules, cell surface receptors, cytokines, growth factors and other proteases (Ethell and Ethell 2007; Vafadari et al. 2016). While MMP-9 levels are lower in the adult brain, its activity has been shown to increase in response to synaptic activity (Gawlak et al. 2009; Janusz et al. 2013).

One of the main mechanisms of MMP-9 activity regulation is via TIMP-1 which is secreted in response to synaptic activity at levels similar to MMP-9 (Ethell and Ethell 2007; Vafadari et al. 2016). While both their expression levels are low during adulthood, they remain functionally relevant as evidenced by the association of the compromised TIMP-1/MMP-9 ratio to various CNS pathologies, including epilepsy, multiple sclerosis and bipolar disorder (Jourquin et al. 2005; Rybakowski et al. 2009; Reinhard et al. 2015). Pro-inflammatory cytokines have been repeatedly linked to abnormal expression of these genes (Jourquin et al. 2005; Okulski et al. 2007; Rybakowski et al. 2009; Berretta 2012).

Our previous studies suggest the P15–P21 rat postnatal development period to be sensitive to the administration of IL-1β (Zubareva et al. 2006, 2013; Trofimov et al. 2014, 2016). In a rat, the myelination and synaptogenesis in the prefrontal cortex and hippocampus peaks during this developmental period (O’Callaghan and Miller 1989; Rice and Barone 2000). In the present study, we investigate whether or not an early-life (P15–P21) pro-inflammatory LPS challenge that closely mimics clinical conditions alters motor learning in rats. As altered hypothalamic–pituitary–adrenal (HPA) axis functions are one of the long-lasting consequences of early-life inflammatory challenge and are known to negatively affect motor learning (Girard-Joyal et al. 2015; Kasahara et al. 2015), we also assessed hormonal and behavioural measures of stress response in postnatally challenged adult rats.

## Material and methods

### Animals

Two-month-old Wistar rats were obtained from a licensed provider, Rappolovo (Leningrad Region, Russia; licensed GOST-R-989112). Animals were housed under standard conditions (see Supporting Information). All studies conformed to the regulations outlined in the European Communities Council Directive (86/609/European Economic Community) and were approved by the ethics authorities of the Institute of Experimental Medicine, St. Petersburg.

### Study Outline

**Fig. 1:**
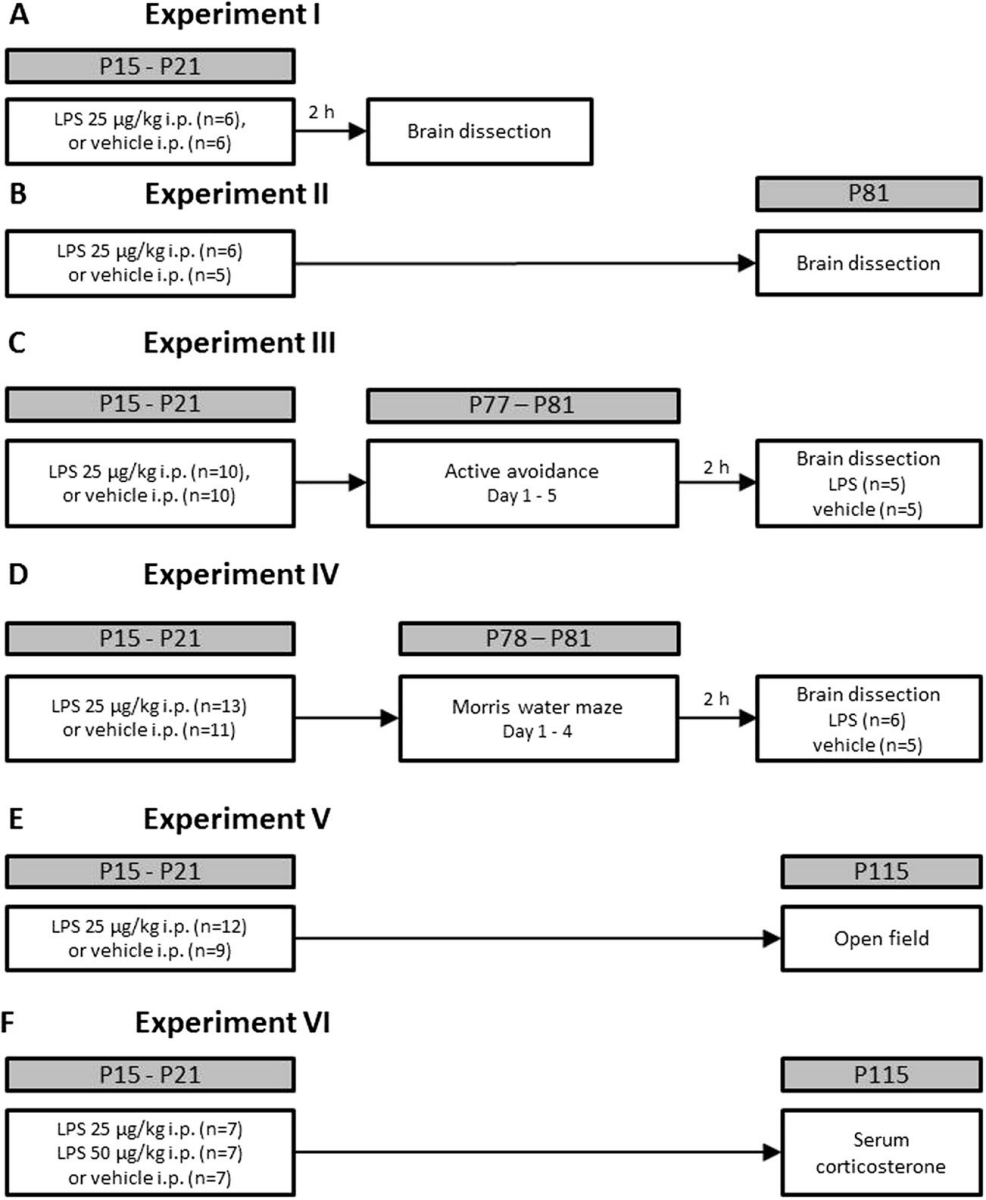
Schematic outline of studies. In each study, rat pups received LPS injections on P15, P18 and P21. Expression of developmental plasticity factors was studied **a** 2 h post-injection (experiment I) and **b** in adulthood (experiment II). The following assays were performed: **c** footshock active avoidance (experiment III), **d** water maze escape learning (experiment IV), **e** open-field behaviour and **f** ELISA of plasma corticosterone

One male and four female rats were co-housed until the females were pregnant, as described elsewhere (Pawluski et al. 2012); thereafter, females were single-housed until the pups were born. Experimental groups were balanced by body weight and received three injections of saline or LPS (25 μg/kg) on days P15, P18, and P21 (Fig. 1a–f). In experiments I and II, LPS-challenged animals were sacrificed 2 h post-injection or on P81. *Mmp9* and *Timp1* were evaluated in the medial pre-frontal cortex (mPFC), dorsal hippocampus (DH) and ventral hippocampus (VH) using RT-PCR. In experiments III and IV, LPS-challenged rats were trained in a 5-day active avoidance footshock or a 4-day water maze task; 2 h after the last session, rats were killed and the above-indicated brain regions dissected for RT-PCR of *Mmp9* and *Timp1* mRNA. For experiments V and VI, LPS-challenged animals’ open-field behaviour or serum was studied; an additional dose of 50 μg/kg of LPS was used in the latter study. Group sizes are indicated in Fig. 1. For details regarding LPS administration, see Supporting Information.

### Behavioural Tests

#### Active Avoidance Task

During five consecutive days, rats were trained to associate light stimulation with a mild footshock. On day 1, they were placed in a customized transparent Plexiglas two-chamber shuttle box (40 × 30 × 55 cm) with grid floor (bars 1 cm apart) and after a 5-min acclimatization period were exposed to a 5-s light stimulation (lighting intensity on grid 110 lx, lamp 10 cm above the grid) followed by a footshock (constant current of 0.5 mA, 1 s) for ten consecutive sessions. During days 2–5, each rat underwent 20 training sessions with random intersession intervals of 20–40 s. The percentage and latency of avoidance responses, defined as rat movement to a shock-free chamber after the conditioned stimulus but prior to shock, were recorded. Escape behaviour as defined by the displacement of a rat to a shock-free chamber after the shock delivery was scored as well.

#### Water Maze

Various A4 sheet size visual cues were placed on the inner walls of the tank (Ø = 150 cm, walls 70 cm high) at positions north-east (NE), south-east (SE), south-west (SW) and north-west (NW). Four times a day on four consecutive days, animals were placed for 90 s in a pool of water mixed with milk, with 90-s inter-trial intervals. A platform (10 × 10 cm) was submerged 1 cm below water, at the centre of the NW sector (Trofimov et al. 2014). The four above-indicated starting points were randomly used as starting points for animals. After a rat reached the platform or was placed there when the trial elapsed, it was left there for 30 s. Previous studies revealed no significant changes in spatial learning but instrumental-like performance under these conditions (Umriukhin and Strekalova, *unpublished data*). The percentage of animals that reached the platform within 90 s (escape response) and the average swimming speed were evaluated using the previously validated video-tracking program “Pavlovian Tracking” (St. Petersburg, Russia).

#### Open Field

Animals were placed at the centre of a black arena (Ø = 100 cm, lighting intensity 5 lx) for 3 min (Veniaminova and Zubareva 2015). The number of rearings and freezing events, defined by the absence of movements besides breathing, were recorded as described elsewhere (Strekalova et al. 2003; Vignisse et al. 2014). Behaviours were scored using the previously validated tracking program “Field4W” (St. Petersburg, Russia).

### Brain Dissection and qRT-PCR

Brain dissection was carried out as described elsewhere (Morozova et al. 2016), and samples were immediately frozen and stored at −70 °C until use. Total RNA was isolated using TRI Reagent (Molecular Research Center Inc., Cincinnati, OH, USA). Two micrograms of total RNA was reverse-transcribed into cDNA by M-MLV reverse transcriptase (Promega Corporation, Madison, WI, USA). Gene expression TaqMan assays were performed for *Timp1*, *Mmp9* and housekeeping gene *Gapdh* on the CFX96 Touch™ Real-Time PCR Detection System (Bio-Rad Hercules, CA, USA). Glyceraldehyde 3-phosphate dehydrogenase (*Gapdh*) was chosen as a reference gene based on our previous results (Couch et al. 2016). For primers and probe sequences (Alcor Bio, St. Petersburg, Russia) and cycling conditions, see Supporting Information. Relative mRNA levels were determined using the cycle threshold (Ct) and the 2^−ΔΔCt^ method as described previously (Couch et al. 2016) for both genes of interest, and the *Timp1:Mmp9* ratio, a marker of developmental plasticity, was calculated (means of groups are in Table 2 of Supporting Information). All data were normalized to the means of respective control groups and expressed as a percentage.

### ELISA of Corticosterone

Blood was collected, and samples of volume 100–200 μl were left at room temperature for 60 min and then centrifuged at 800×*g* for 10 min at room temperature. Serum was collected and analysed for corticosterone concentration with a DRG Corticosterone ELISA Kit (DRG International Inc., East Mountainside, MG, USA) in accordance with instructions of the manufacturer on a Microplate Reader Immunochem-2100 (HTI Diagnostics, Walpole, MA, USA).

### Statistical Analysis

Data were analysed in Prism6.0 (GraphPad Software, Inc., USA). Two-group comparisons of gene expression and learning assays were performed using Mann–Whitney *U* test, as these data did not pass the Shapiro–Wilk test for normal distribution. A three-group comparison of the normally distributed data from the ELISA study was carried out using ANOVA and Tukey’s post hoc test. Linear regression was used to perform correlation analysis. Statistical significance was set at *p* < 0.05. Data are shown as mean ± SEM.

## Results

### Postnatal Administration of LPS Induces Differential Changes in Timp1 and Mmp9 in Pups versus Adult Rats and the Study of Escape Task Training in Adulthood

**Fig. 2:**
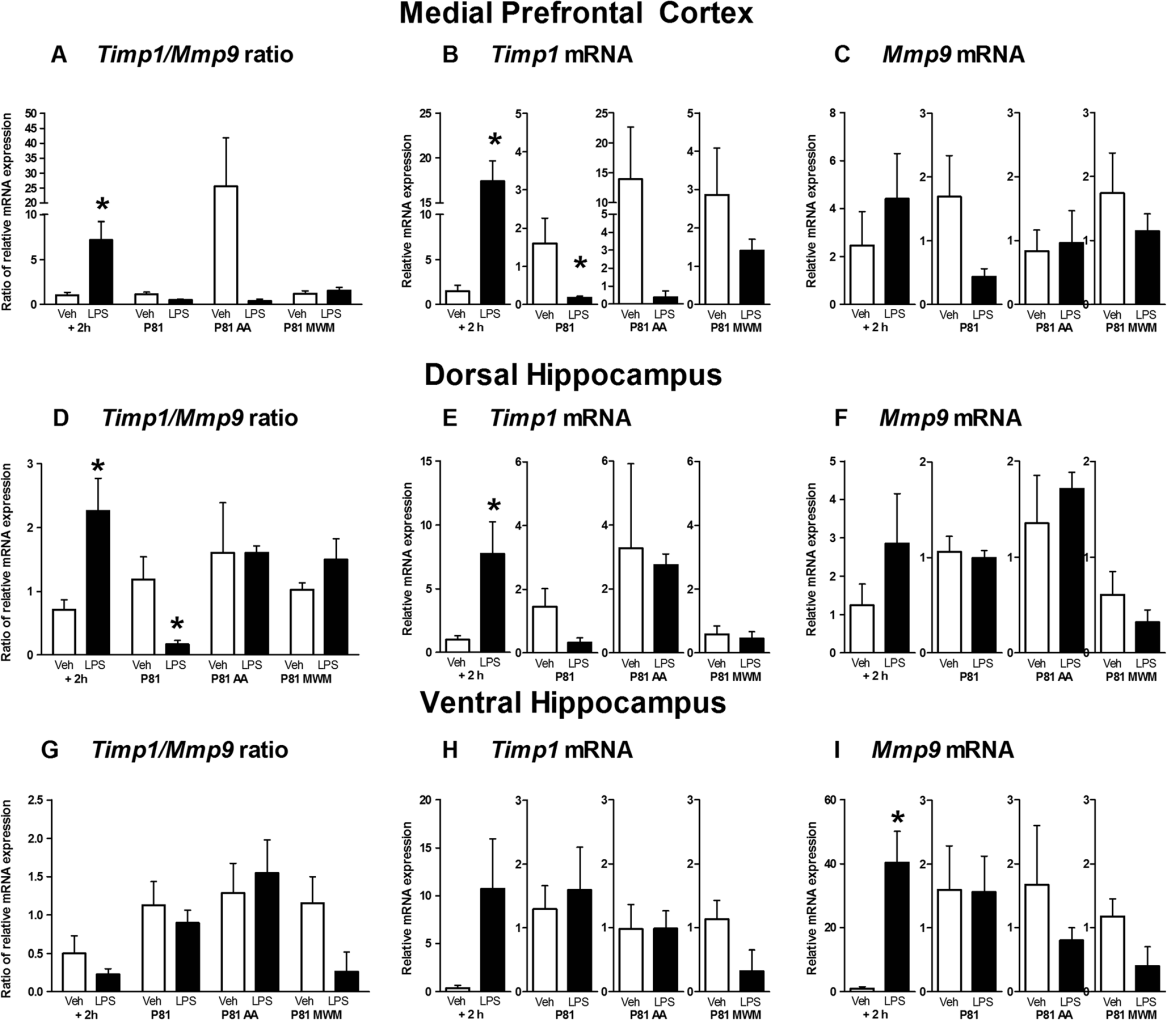
Postnatal LPS administration differentially affects brain expression of *Timp1* and *Mmp9* in pups and adult untrained rats. All comparisons are normalized to vehicle-treated controls. In the mPFC, **a** the LPS-challenged pup *Timp1:Mmp9* ratio was significantly higher, while adult LPS-treated, untrained rats had a tendency to a decreased ratio. **b** *Timp1* levels were significantly elevated in LPS-treated pups and decreased in LPS-challenged adult rats, and **c** there were no significant changes in *Mmp9* in LPS-treated rats; adult LPS-treated rats trained in active avoidance or water maze tests had unaltered gene expression. In the DH, **d** LPS-treated pups showed significantly higher *Timp1:Mmp9* ratio, whereas adult untrained rats had opposite changes. **e** *Timp1* was significantly elevated in LPS-treated pups, while adult rats showed a tendency to reduced *Timp1*. **f** No significant changes were found in *Mmp9* in the LPS-challenged groups; rats subjected to training showed unaltered gene expression. In the VH, LPS-treated pups had a non-significant decrease of **g** *Timp1:Mmp9* ratio, **h** a non-significant increase of *Timp1* and **i** a significant increase of *Mmp9* levels. *p < 0.05 versus control. *Veh* vehicle-treated controls; *LPS* LPS-challenged; *AA* active avoidance-trained rats; *WM* water maze-trained rats. See the text

In the mPFC, LPS-challenged pups had a significantly higher *Timp1:Mmp9* ratio whereas naive LPS-challenged rats showed a tendency towards a reduced ratio compared with the vehicle-treated control pups (*U* = 0.0, *p =* 0.016, Mann–Whitney *U* test; *U* = 0.0, *p =* 0.095; respectively; Fig. 2a; all means of relative fold expression data are presented in Supporting Information, Table 2). In comparison to control rats, *Timp1* levels were significantly increased in LPS-challenged pups (*U* = 0.0, *p =* 0.01; Fig. 2b) and significantly reduced in LPS-challenged adult rats (*U* = 2.0, *p =* 0.032; Fig. 2b). Adult LPS-challenged rats subjected to active avoidance or water maze training had a non-significant decrease of both *Timp1:Mmp9* ratios and *Timp1* levels relative to controls (*U* = 8.5, *p =* 0.46 and *U* = 7.0, *p =* 0.556; respectively; Fig. 2b). None of the LPS-challenged groups displayed altered *Mmp9* levels in comparison to control rats, while a non-significant optical decrease in expression levels was seen in the naïve LPS-treated rats (pups: *U* = 11.0, *p =* 0.528; untrained adults: *U* = 8.0, *p =* 0.389; adult active avoidance: *U* = 11.0, *p =* 0.802; adult water maze: *U* = 10.0, *p =* 0.635; Fig. 2c).

As for the DH, LPS-challenged pups had significantly higher *Timp1:Mmp9* ratios compared to controls while LPS-challenged untrained adults showed reduced ratios (*U* = 2.0, *p =* 0.017; *U* = 0.0, *p =* 0.008; respectively; Fig. 2d). Active avoidance or water maze paradigm-trained rats showed no ratio changes compared with controls (*U* = 7.0, *p =* 0.310; *U* = 7.0, *p =* 0.310; respectively; Fig. 2d); the latter group displayed a non-significant decrease of this measure. LPS-challenged pups had significantly increased *Timp1* in the DH (*U* = 0.0, *p =* 0.004) with a contrasting non-significant reduction of *Timp1* in adult LPS-challenged untrained rats and rats trained in active avoidance learning (*U* = 5.0, *p =* 0.171 and *U* = 5.0, *p =* 0.151; respectively; Fig. 2e). No changes were found in LPS-challenged adult rats exposed to the water maze task (*U* = 6.0, *p =* 0.686; Fig. 2e). No significant changes in the *Mmp9* levels were found in any LPS-challenged rat groups in comparison to their respective vehicle-treated controls (pups: *U* = 7.0, *p =* 0.310; adults: *U* = 8.0, *p =* 0.397; adult active avoidance: *U* = 9.0, *p =* 0.532; adult water maze: *U* = 5.0, *p =* 0.486; Fig. 2f).

Finally in the VH, LPS-challenged pups showed a significant increase of *Mmp9* (*U* = 1.0, *p =* 0.019; Fig. 2i) with a non-significant reduction in the *Timp1:Mmp9* ratio (*U* = 8.0, *p* = 0.474; Fig. 2g) and a non-significant elevation of *Timp1* (*U* = 8.0, *p* = 0.448; Fig. 2h) relative to the respective control group. In LPS-challenged adult rats, no differences from controls were found in the *Timp1:Mmp9* ratio in the VH (untrained adults: *U* = 11.0, *p* = 0.895; adult active avoidance: *U* = 10.0, *p* = 0.667; adult water maze, *U* = 3.0, *p* = 0.109; Fig. 2g). Between these groups, no significant differences were found in *Timp1* in the VH (untrained adults: *U* = 12.0, *p* = 1.000; adult active avoidance: *U* = 11.0, *p* = 0.847; adult water maze, *U* = 4.0, *p* = 0.168; Fig. 2h). Similarly, no significant differences were found in Mmp9 in the VH (untrained adults: *U* = 11.0, *p* = 0.914; adult active avoidance: *U* = 10.0, *p* = 0.690; adult water maze, *U* = 3.0, *p* = 0.111; Fig. 2i). Thus, LPS administration in the early postnatal period resulted in aberrations of the TIMP1/MMP9 regulatory system across several regions of the developing limbic system, with directionally opposite changes in adulthood, which were not that evident in rats subjected to active avoidance and water maze.

### Postnatal Administration of LPS Results in Deficient Acquisition of Avoidance Task in the Adulthood

**Fig. 3:**
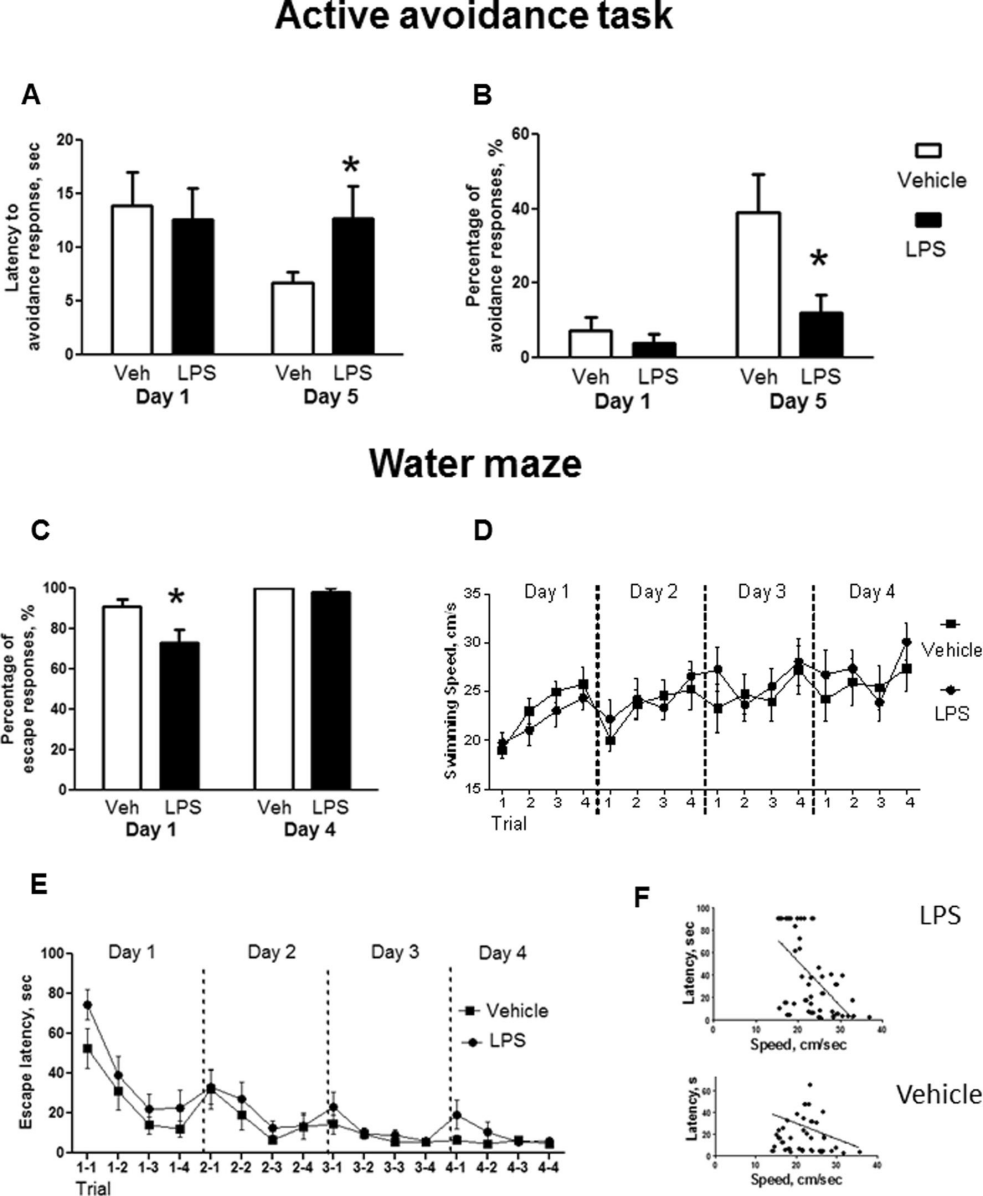
Postnatal LPS treatment affects the acquisition of escape abilities in two memory paradigms. In the active avoidance task, LPS-challenged rats showed **a** longer latencies of avoidance response and **b** smaller percentage of avoidance responses than vehicle-treated rats, on day 5 but not day 1. In the water maze, in comparison with controls, LPS-treated rats showed **c** a decreased percentage of avoidance responses on day 1 but not day 4 of training. LPS- and vehicle-injected animals showed similar **d** swimming speed and **e** mean escape latency. **f** In both LPS- and vehicle-treated rats, there was a significant correlation between swimming speed and escape latency. *p < 0.05 versus controls. Abbreviations are as in Fig. 2

On day 5, LPS-challenged rats showed significantly longer latency and a reduced percentage of avoidance responses than controls (*U* = 23.0, *p =* 0.043; *U* = 23.5, *p =* 0.042, respectively; Fig. 3a, b). The latency and percentage of avoidance responses on days 1–4 of the active avoidance model were not significantly different between LPS-challenged and control animals (day 1: *U* = 47.0, *p =* 0.836; *U* = 47.5, *p =* 0.777, respectively; Fig. 3b; for days 2–4, see Supporting Information, Figs. 1 and 2). During all trials conducted in the experiment, either avoidance or escape behaviours were displayed by every rat, suggesting similar motor abilities and motivation across LPS- and non-LPS-challenged groups. As such, reduced avoidance memory as found in the LPS-treated animals is not likely to be due to an impairment other than in associative learning.

LPS-challenged rats had a smaller percentage of escape responses on day 1 of the water maze in comparison with controls (*U* = 38.5, *p =* 0.049; Fig. 3c). No differences were observed on day 4 (*U* = 66.0, *p =* 0.999, respectively; Fig. 3c) and days 2–3 (see Supporting Information, Fig. 3). The swimming speed of LPS-exposed rats did not differ from controls (Fig. 3d). Also, we found that the mean escape latencies were not significantly different between the groups at any time point during the experiment (*p* > 0.05; Fig. 3e). Both vehicle- and LPS-challenged groups showed a significant correlation between the mean escape latency and the mean speed of swimming (*r* = 0.29, *p* = 0.02 and *r* = 0.58, *p* = 0.001, respectively; Fig. 3f). These data suggest a mild deficiency in motor tasks in LPS-challenged rats and rule out the possibility that other general factors not related to learning abilities impact the acquisition of escape responses in this assay.

### Altered Freezing Behaviour and Basal Plasma Corticosterone Levels in Adult Rats Subjected to Postnatal Administration of LPS

**Fig. 4:**
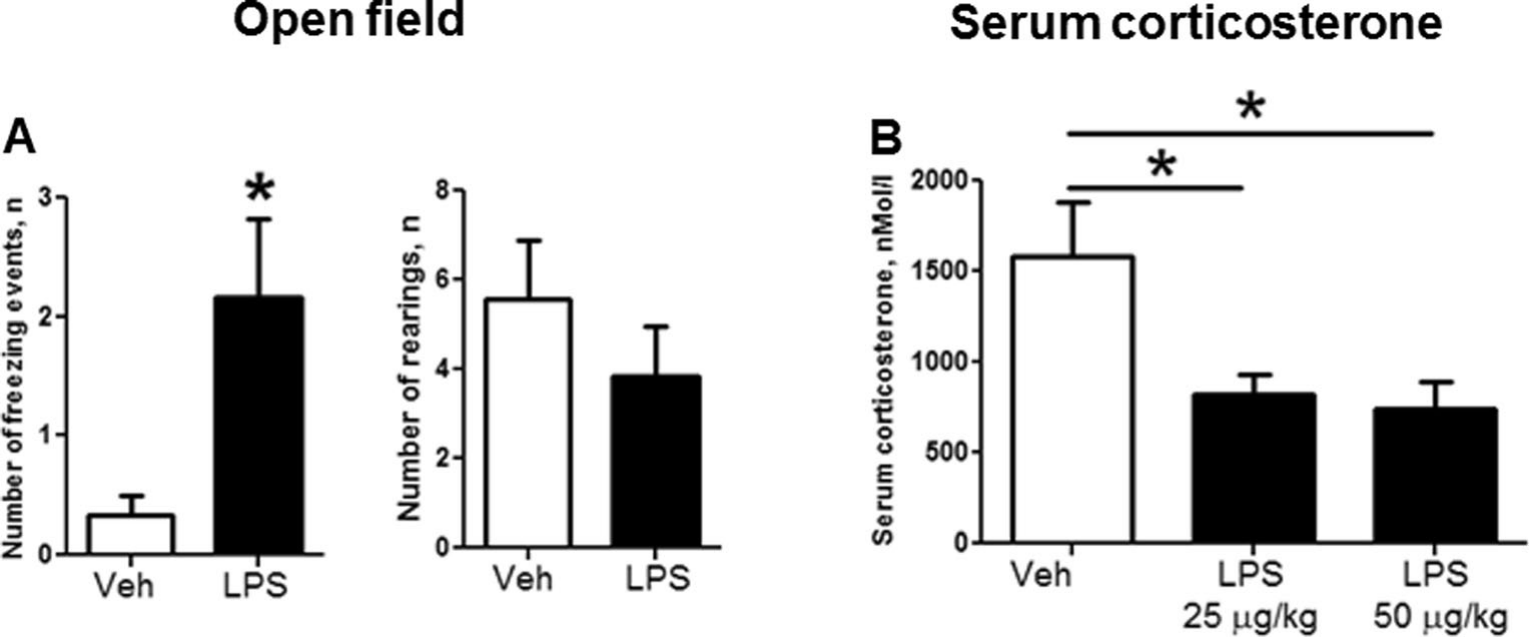
Postnatal LPS administration alters stress-related features. In the open-field test, as compared to controls, LPS-treated adult rats showed **a** a significantly elevated number of freezing events and **b** no changes in rearing activity. **c** Adult LPS-treated rats had significantly decreased basal levels of serum corticosterone in comparison with control rats *p < 0.05 versus control animals. Abbreviations are as in Fig. 2

In the open-field test, in comparison to control animals, LPS-challenged rats had more freezing events (*U* = 25.5, *p =* 0.032) and similar number of rearings as controls (*U* = 38.0, *p =* 0.272; Fig. 4b), suggesting that the measures in locomotor activity are not related to the above-described group differences in learning scores. Postnatal challenge with LPS at a dose of 25 or 50 μg/kg resulted in decreased basal level of plasma corticosterone in comparison with controls; no significant difference was found between the two LPS dosage groups (*F*_(2,18)_ = 4.997, *p =* 0.019, ANOVA; LPS 25 and 50 μg/kg: *q* = 3.666, *p =* 0.04; *q* = 4.049, *p =* 0.027 versus control; *q* = 0.383, *p =* 0.960, respectively; Tukey test, Fig. 4c).

## Discussion

In this study, we found persisting effects of postnatal systemic inflammatory challenge, on escape learning in the footshock-elicited active avoidance and water maze paradigms and on the expression of developmental plasticity factors *TIMP-1/MMP-9* in brain regions regulating emotionality and memory. These data suggest that early-life inflammation affects evolutionally ancient forms of learning, such as the acquisition of motor escape abilities, which are generally known to be robust under various pathological conditions. In our study, early life immune challenge of rats with LPS resulted in increased freezing behaviour and unexpectedly diminished serum corticosterone levels in adulthood, suggesting altered mechanisms of stress response. A lack of significant molecular aberrations found in animals subjected to both postnatal LPS challenge and repeated escape training could be of potential functional importance.

Here, we report an increase in brain *Timp1* and unchanged *Mmp9* expression in LPS-challenged pups, which may shift the balance between these factors and impact MMP-9, an important regulator of morphological and synaptic plasticity (Bozdagi et al. 2007; Wlodarczyk et al. 2011). MMP-9 has been shown to be involved in the modification of dendritic spines during neuronal stimulation and dendrite growth (Wang et al. 2008; Bilousova et al. 2009) possibly via regulation of the degradation of intercellular adhesion molecule (ICAM)-5 (Tian et al. 2007) and integrin-β1-mediated signalling (Michaluk et al. 2011) and by increasing the lateral mobility of the NMDA receptors (Michaluk et al. 2011). In particular, incubation with MMP-9 changes the morphology of dendritic spines from a “more mature” mushroom-like form to a “less mature” filopodia-like form in a culture of neurons (Bilousova et al. 2009). Both decreases in MMP-9 expression and increases in TIMP-1 expression are related to the inhibition of LTP in the hippocampus. The development of the late phase of LTP was associated with increases of the concentration and proteolytic activity of MMP-9, while the inactivation of MMP-9 impaired LTP in the CA3-CA1 regions of hippocampal slices (Nagy et al. 2006). In vivo experiments have confirmed the role of MMP-9 in LTP induction and maintenance (Bozdagi et al. 2007). At the same time, TIMP-1 overexpression was shown to impair hippocampal LTP (Okulski et al. 2007). Given that synaptic remodelling and LTP induction are well-established parallels of learning abilities in rodent models, a dysregulation of TIMP-1/MMP-9 expression could underpin aberrant cognitive scores of LPS-challenged rats in our study.

The directions of molecular alterations induced by postnatal LPS injections were opposing in pups and adults, suggesting that potential compensatory processes occur in the TIMP-1/MMP-9 system in adulthood. The occurrence of compensatory changes in the TIMP-1/MMP-9 pathway during adulthood supports its functional importance in the mature brain as previously suggested by clinical observations (Docherty et al. 1992; Bednarek et al. 2012). Two-wave changes in the expression of brain plasticity factors after postnatal inflammatory challenges have been reported previously: for example, LPS injection at P5 initially increased expression of the NMDA receptor NR1 subunit followed by a decrease in adulthood (Harré et al. 2008). Here, early postnatal challenge with LPS resulted in an increase in *Timp1* expression which has been previously linked to reduced synaptic plasticity (Okulski et al. 2007). In contrast, adult animals which were postnatally injected with LPS showed a decrease in *Timp1* expression and *Timp1/Mmp9* ratio in the present study. *Timp1* and *Timp1/Mmp9* ratio reductions have been shown to improve synaptic plasticity, as discussed above (Nagy et al. 2006; Bozdagi et al. 2007). Since this group of rats displayed aberrant learning abilities, these deficits are likely to be due to alternative, possibly developmental, TIMP-1/MMP-9-related mechanisms.

In the current study, molecular changes diverged between the investigated brain structures and seemed to be more pronounced in the mPFC and DH than in the VH. These differences are potentially a result of the different roles of these brain areas in mechanisms of learning versus stress response (Bagot et al. 2015) and the structure-specific effects of LPS that have been reported (Dinel et al. 2014). Previous studies demonstrated greater negative effects of LPS on neuronal survival and plasticity of dorsal versus ventral hippocampus (Järlestedt et al. 2013). Conversely, early-life stress predominantly affected plasticity of the ventral hippocampus and prefrontal cortex, but not dorsal hippocampus (Maras et al. 2014; Calabrese et al. 2015). Overly, all investigated brain areas have revealed expression changes in the TIMP-1/MMP-9 pathway which leads us to speculate that both mechanisms of plasticity and stress are affected in adult rats prenatally exposed to systemic inflammation.

Our study reveals short- and long-lasting effects of systemic inflammation on plasticity factors, which are particularly important during development and have so far been addressed in a very limited number of studies. This approach of identifying developmental molecules vulnerable to systemic inflammation rather than those known to be involved in adult brain plasticity could be of greater importance to furthering our understanding of the primary mechanisms through which early-life stress can lead to impairments in adult brain functions.

Postnatally LPS-challenged rats exposed to the active avoidance or water maze learning showed no significant changes in the expression of *Timp1* or *Mmp9*, while similar to the untrained LPS-treated group, a non-significant movement towards compromised *Timp1* and *Timp1/Mmp9* ratio in the prefrontal cortex was observed in rats subjected to active avoidance. LPS-challenged rats trained in the water maze task have shown an optical reduction of all three parameters in ventral hippocampus; however, the differences in expression levels in the untrained LPS-treated group were far from significant. A lack of significant changes in the investigated plasticity factors in repeatedly trained LPS-challenged rats could be interpreted as a consequence of normalizing synaptic remodelling which is a well-established effect of chronic training in various memory tasks (Pereira et al. 2007; Stamatakis et al. 2014; Smolen et al. 2016). At the same time, the large variability and low levels of *Timp1* and *Mmp9* found in this study, as is characteristic of the expression of developmental factors during adulthood, limit the strength of this hypothesis.

Early-life inflammatory challenge was found in our work to result in deficient motor learning of active avoidance and water maze tasks. Up to now, the majority of literature regarding the effects of postnatal systemic inflammation on brain plasticity predominantly reported changes in hippocampus-dependent learning. However, several observations are in line with our results and suggest that postnatal inflammation can affect other forms of memory as well. Postnatal LPS injection impairs object recognition memory (Hennigan et al. 2007), spontaneous alteration and working memory in the T maze (Hauss-Wegrzyniak et al. 1998), while high-dosage LPS disrupts instrumental learning of the instrumental flexion response (Young et al. 2007). Thus, early-life systemic inflammation affects not only evolutionarily novel types of memory, which are known to be particularly vulnerable under various pathological conditions (Vereker et al. 2000; Rachal Pugh et al. 2001; Shaw et al. 2001), but also more basic forms of learning, such as the acquisition of motor escape abilities, which are generally less disrupted by deleterious factors.

In the present study, LPS-challenged rats showed deficient learning at the beginning of the water maze training, while in the active avoidance paradigm, memory deficits occurred at the end of the experiment. Based on the above-discussed behavioural and gene expression assay results, it can be speculated that in repeatedly trained LPS-challenged rats, the dynamics of learning deficits parallel the altered expression of developmental plasticity genes along with other unknown functional changes. The different time points at which deficits of acquisition occur in the applied memory tests may be due to the different roles of the hippocampus and prefrontal cortex, investigated plasticity factors and HPA-related mechanisms, in the two rodent models of learning.

For example, previous studies have revealed a link between neophobia and deficits in motor learning and other forms of memory (Hernadi et al. 1997; Strekalova et al. 2013; Sarowar et al. 2016), including the water maze task (Li et al. 2002; Kelly et al. 2003). While the factor of neophobia could be substantial during the first sessions of the water maze training of LPS-treated rats, it is unlikely to have played a significant role at later training sessions, nor during the active avoidance paradigm, where animals were exposed to pre-training habituation. In the latter paradigm, however, a stress impact of training might be of importance, since repeated footshock is well known to induce stress response in rodents particularly, affecting glucocorticoids (Rosecrans et al. 1986; D’Hooge and De Deyn 2001). Altered neophobic responses and blood corticosterone levels are known to involve HPA-related mechanisms, the change of which is one of the major features of early-life systemic inflammation (Shanks et al. 1995; Ellis et al. 2006; Dinel et al. 2014).

Evidence for altered HPA activity was found in the present work. Our study revealed increased freezing behaviour in postnatally LPS-challenged rats tested under mild lighting conditions in a novel open-field situation, where anxiety-like behaviours are normally not evoked in rodents (Strekalova et al. 2005; Strekalova and Steinbusch 2010). Previous studies that employed open-field testing with similar illumination strength have demonstrated behavioural abnormalities in mice and rats with altered HPA activity (Couch et al. 2013; Pawluski et al. 2012). Hence, our present findings suggest an increased HPA responsiveness to stress in postnatally LPS-challenged rats. Indeed, various aberrations in neuronal functions at adulthood associated with early-life systemic inflammation are considered to result from elevated HPA axis activity. Inflammatory activation of the HPA axis was shown to provide an important regulatory feedback to the pro-inflammatory cytokines that limits their synthesis (Besedovsky et al. 1986; Del Rey et al. 1987). In rats, exposure to LPS in early life increases corticotrophin-releasing hormone expression in the hypothalamus, decreasing glucocorticoid receptor density in the hypothalamus, hippocampus and frontal cortex (Shanks et al. 1995) and affecting its phosphorylation (Dinel et al. 2014).

While most previous studies carried out on adults report increased corticosterone levels after systemic inflammatory challenge (Kohman et al. 2008; Kasahara et al. 2015; Girard-Joyal et al. 2015), we found that early-life administration of two different doses of LPS produced a prominent decrease in corticosterone, suggesting compensatory changes in the HPA system. Various challenges that normally increase HPA activity and corticosterone levels in adulthood (Maccari et al. 2003; Weinstock 2008) have been shown to have no effect (Koenig et al. 2005; Weinstock 2008; Dinel et al. 2014) or, like in our study, result in decreases (Cannizzaro et al. 2006; Solati et al. 2015) when applied during development. One of the explanations for the paradoxical decrease of plasma corticosterone in our work can be due to the previously discussed over-expression of hypothalamic hormones regulating adrenal function after early-life LPS injection, which may be followed by a compensatory suppression of glucocorticoid production (Shanks et al. 1995; Dinel et al. 2014). The direction of corticosterone changes in rodents postnatally treated with LPS was shown to be linked to the development period (Dinel et al. 2014; Girard-Joyal et al. 2015). For instance, the effects of LPS on blood corticosterone levels in mice were significantly weaker at the peripubertal/adolescent period, particularly in males.

Together, our data concerning increased freezing behaviour and decreased plasma corticosterone levels suggest that early-life systemic inflammation generates long-lasting alternations in stress response and HPA axis functions. The long-lasting impact of an early-life immune challenge on HPA responsiveness is linked to its tight relationship with neuroimmune mechanisms (Spencer et al. 2006; Dinel et al. 2014). HPA axis activation in animals can be altered by early-life LPS activation which may result in abnormal triggering of the complex regulatory responses of stress hormones to environmental factors (Besedovsky et al. 1986; Mouihate et al. 2010). Such an abnormality in response could occur in response to the novelty stress which rats are exposed to during our open-field paradigm, which might evoke the paradoxical effect of a suppression of corticosterone release.

In summation, postnatal systemic inflammation compromises motor escape learning in adulthood, accompanied by aberrant expression of the *TIMP-1/MMP-9* developmental plasticity pathway. These abnormalities, along with alterations in both hormonal and behavioural markers of stress response, suggest that the learning deficits associated with early-life LPS challenge may arise as a result of disruptions to the plasticity-associated TIMP-1/MMP-9 cascade and aberrant HPA activity.

## Electronic supplementary material

ESM 1 (DOCX 125 kb).
