# Supplementary material for: Postnatal LPS Challenge Impacts Escape Learning and Expression of Plasticity Factors Mmp9 and Timp1 in Rats: Effects of Repeated Training
Source: Neurotox Res. 2017 Apr 18;32(2):175–86. doi: 10.1007/s12640-017-9720-2 (PMC5493723; doi:10.1007/s12640-017-9720-2)
Supplement: Supplementary file 1 — (DOCX 125 kb). [file 12640_2017_9720_MOESM1_ESM.docx]

**Supporting Material**

**Animals**

We used Wistar rats, that were 2 month-old and obtained from “Rappolovo”, a provider licensed by Charles River (Leningrad Region, Russia; licensed GOST R 989112), for breeding. Animals were housed under standard conditions (see Supporting Material) in standard plastic cages (42x26x15 cm) under standard laboratory conditions (21 ± 2 °C, 65% humidity, with food and water ad libitum) with the 12:12 h light-dark cycle, lights on at 10:00 a.m. All studies were carried out in accordance with the European Communities Council Directive (86/609/European Economic Community) and were approved by the local biomedical ethics committee of the Institute of Experimental Medicine. All efforts were undertaken to minimize the potential discomfort of experimental animals.

**Drug administration**

On P15, P18 and P21, rat pups were exposed to single LPS administration. LPS (*E.coli* 055:B5, Sigma-Aldrich, St. Louis, MO, USA) was dissolved in sterile saline (0.9 %) and injected intraperitoneally (i.p.) at the doses 25µg/kg or 50µg/kg (see manuscript text) in a volume of 1 ml of the solution per every 10 g of animal weight. Control animals received i.p. injections of vehicle.

***Table 1. Sequences of primers used***

| **Gene** | **Forward primer 5′–3′** | **Reverse primer 5′–3′** | **Probe 5′–3′** |
| --- | --- | --- | --- |
| *Gapdh* | tgcaccaccaactgcttag | ggatgcagggatgatgttc | hex-atcacgccacagctttccagaggg-bhq1 |
| *Mmp9* | cctctgcatgaagacgacat | gaggtgcagtgggacacata | rox-ctgtatggtcgtggctctaaacctga-bhq2 |
| *Timp1* | ctggcataatctgagccctg | gcaaagtgatcgctctggtag | fam-tgtgcacagtgtttccctgttcagc-bhq1 |

**RNA isolation and RT PCR**

The qRT-PCR was performed in a 12 μl reaction volume containing a 10хPCR Buffer (1.2 μl), 25 mmol MgCl_2_ (2 μl), 10 mmol dNTPs (1 μl), specific forward and reverse primers at 20 pmol/μl concentration (0.5 μl), 10 pmol/μl probe (0.3 μl), cDNA (1 μl), 5 u/μl Taq DNA polymerase (0.5 μl) (Beagle, St. Petersburg, Russia), and ddH_2_O (5 μl). All samples were run in triplicate. Cycling was performed at 95 C° for 5 min followed by a 50-cycle amplification at 95°C for 5 s, then at the annealing temperature 60°C defined previously for 10 s and at the temperature 72°C for 15 s.

***Table 2. Changes in fold expression of Mmp9 and Timp1 plasticity factors in three brain regions*** (see the manuscript text)

| **Brain structure** |  | **Median Prefrontal Cortex** | | **Dorsal Hippocampus** | | **Ventral Hippocampus** | |
| --- | --- | --- | --- | --- | --- | --- | --- |
| **Gene/ time point** |  | Timp1 | Mmp9 | Timp1 | Mmp9 | Timp1 | Mmp9 |
| +2 h | Vehicle | **1.50 ± 0.63**** | 2.47 ± 1.41 | **0.99 ± 0.32**** | 1.25 ± 0.55 | 0.40 ± 0.26 | **0.94 ± 0.66*** |
|  | LPS | **17.40 ± 2.30**** | 4.41 ± 1.89 | **7.73 ± 2.53**** | 2.85 ± 1.30 | 10.73 ± 5.20 | **40.43 ± 9.78*** |
| P81 | Vehicle | **1.60 ± 0.66*** | 1.69 ± 0.65 | 1.46 ± 0.57 | 1.06 ± 0.16 | 1.30 ± 0.37 | 1.60 ± 0.69 |
|  | LPS | **0.18 ± 0.04*** | 0.43 ± 0.13 | 0.33 ± 0.15 | 1.00 ± 0.08 | 1.60 ± 0.68 | 1.56 ± 0.56 |
| P81 AA | Vehicle | 13.90 ± 8.75 | 0.84 ± 0.33 | 3.27 ± 2.64 | 1.36 ± 0.50 | 0.98 ± 0.39 | 1.68 ± 0.93 |
|  | LPS | 0.39 ± 0.34 | 0.97 ± 0.50 | 2.76 ± 0.34 | 1.71 ± 0.17 | 0.98 ± 0.28 | 0.80 ± 0.20 |
| P81 MWM | Vehicle | 2.86 ± 1.23 | 1.74 ± 0.63 | 0.58 ± 0.26 | 0.61 ± 0.24 | 1.13 ± 0.30 | 1.17 ± 0.27 |
|  | LPS | 1.40 ± 0.30 | 1.15 ± 0.27 | 0.45 ± 0.21 | 0.32 ± 0.13 | 0.33 ± 0.33 | 0.40 ± 0.30 |

**Figure 1**

**Figure 1. Latency to avoidance response in active avoidance task of rats postnatally challenged with LPS.** Averaged latency to avoidance response calculated across all training sessions on Days 2-4 was non-significantly longer in LPS-challenged rats than in control animals (Mann-Whitney, p>0.05). This difference was significant on Day 5 (Mann-Whitney, p<0.05, see the manuscript text).

**Figure 2**

**Figure 2. Percentage of avoidance responses in active avoidance task of rats postnatally challenged with LPS.** Averaged percentage of avoidance responses calculated across all training sessions on Days 1-4 was non-significantly lower in LPS-challenged rats than in control animals (Mann-Whitney, p>0.05). This difference was significant on Day 5 (Mann-Whitney, p<0.05, see the manuscript text).

**Figure 3**

**Figure 3. Percentage of escape responses in a water-maze of rats postnatally challenged with LPS.** Averaged percentage of escape responses calculated across all training sessions on Days 1 was significantly smaller in LPS-challenged rats than in control animals (Mann-Whitney, p<0.05, see the manuscript text). This difference was non-significant on Days 2-4 (Mann-Whitney, p>0.05).
